# Management dilemma; a woman with cystic fibrosis and severe lung disease presenting with colonic carcinoma: a case report

**DOI:** 10.1186/1752-1947-2-384

**Published:** 2008-12-15

**Authors:** Andrea N Lees, David W Reid

**Affiliations:** 1 Department of Respiratory Medicine, Royal Hobart Hospital, 48 Liverpool Street, Hobart, Tasmania 7000, Australia

## Abstract

**Introduction:**

There are increasing reports of bowel cancer in cystic fibrosis, suggesting a possible causal link. Individuals with cystic fibrosis who have advanced lung disease present a high operative risk, limiting curative treatment options in early bowel malignancy.

**Case presentation:**

We describe a 41-year-old Caucasian woman with cystic fibrosis and severe lung disease who had been considered for lung transplantation, who presented with rectal bleeding and was found to have a Stage I adenocarcinoma of the sigmoid colon. After considerable discussion as to the operative risks, she underwent a laparoscopic resection and remains relatively well 1 year postoperatively with no recurrence.

**Conclusion:**

We discuss the complexity of the management decisions for cystic fibrosis patients with severe lung disease and early stage colonic malignancy, particularly in the context of potential need for lung transplantation. The case demonstrates that cystic fibrosis patients with very severe lung function impairment may undergo laparoscopic abdominal surgical interventions without compromising postoperative airway clearance.

## Introduction

Cystic fibrosis (CF) is the commonest lethal genetic disease to affect Caucasian populations. Death usually results from lung destruction secondary to chronic airway sepsis, but survival has increased over the past three decades and it is predicted that 90% of children born with CF will now survive into their fourth decade. With increasing age, new manifestations of CF have emerged, including an apparently increased risk of gastrointestinal malignancies [1,2], although cases remain rare and less than 25 large bowel malignancies have been described worldwide [3,4]. The youngest reported case was 13 years old, but most occur in the 3rd and 4th decades [3,5].

## Case presentation

A 41-year-old woman with CF was admitted for a course of intravenous antibiotics after developing worsening respiratory symptoms of increased cough, sputum volume and purulence and worsening breathlessness on exercise. She had lost 3 kg in weight and felt generally unwell. Her FEV_1 _had fallen from 860 mL to 780 mL. During the course of her admission, she volunteered that she had noted intermittent rectal bleeding and that the blood appeared to be mixed in with her stools.

The patient's CF genotype was DF508/N1303K. She had severe lung disease and was chronically infected with mucoid *Pseudomonas aeruginosa*. She was pancreatic insufficient and had CF-related diabetes (CFRD), as well as mild CF-related biliary cirrhosis. Treatment when stable consisted of rotating oral and nebulised antibiotics, nebulised DNAse and hypertonic saline, pancreatic enzyme replacement, fat-soluble vitamin supplements and insulin.

The question of lung transplantation had been raised previously and the patient had undergone formal assessment for this in 2005, but following discussions with the transplant team had decided to delay listing because lung function, albeit very poor, had remained stable over the preceding 5 years and her quality-of-life was still reasonable.

Examination revealed a woman at the lower limit of the healthy weight range (BMI 20.2). She was clubbed but there were no signs of anaemia or stigmata of chronic liver disease. Scattered inspiratory crepitations were present throughout both lung fields, especially over the upper lobes, but these findings were unchanged from previous recordings. On abdominal examination, there was no tenderness or palpable masses. Rectal examination was normal. Initial investigations revealed a normal haemoglobin, renal function and serum amylase. Liver enzymes were normal except for a slightly raised serum alkaline phosphatase at 155 IU/L (normal range 45 to 115 IU/L). Chest radiograph showed over-inflated lungs with fibrotic scarring and ring shadows, particularly in the upper lobes, consistent with her advanced lung disease. Full lung function testing showed an FEV_1 _of 0.78 L (29% predicted), forced vital capacity (FVC) of 1.29 L (41% predicted), and evidence of gas trapping with a residual volume of 210% predicted as well as a reduced carbon monoxide transfer factor (TLCO) of 12.86 mL/min/mmHg (52% predicted) that normalised when corrected for alveolar volume.

At colonoscopy, a large pedunculated polyp was seen in the distal sigmoid colon and the top of this was removed. Histopathology demonstrated a moderately differentiated adenocarcinoma arising on a background of a severely dysplastic tubulovillous adenoma. Invasive tumour was apparent at the surgical resection margin. There was no immunohistochemical evidence of mutation in the mismatch repair genes MLH1, MSH2 and MSH6.

A staging computed tomography (CT) scan with contrast of the chest, abdomen and pelvis did not demonstrate the primary malignancy or any metastases within the abdomen.

After a second unsuccessful attempt at endoscopic resection, the patient elected to undergo potentially curative laparoscopic resection. She was transferred to a large tertiary referral hospital in another state that has a fully staffed multidisciplinary CF Unit providing care to over 220 patients. Tasmanians do not have access to this sort of dedicated care team and it was thought that the patient's chances of survival postoperatively would be increased if 24-hour access to a multidisciplinary CF team was available.

As part of the pre-operative staging, a positron emission tomography (PET) scan was undertaken and this demonstrated increased uptake in enlarged mediastinal lymph nodes which were thought reactive and consistent with her chronic pulmonary sepsis. There was no uptake in the abdomen to suggest loco-regional metastatic disease. A laparoscopic anterior resection was performed under general anaesthetic. The procedure was tolerated remarkably well. Operative time was 4 hours during which she maintained oxygen saturations between 97% and 100% on a FiO_2 _of 38%. She was extubated successfully and had an uncomplicated postoperative course. She rapidly weaned herself off a fentanyl infusion (Patient Controlled Analgesia) within 24 hours and was able to undertake chest physiotherapy and airway clearance techniques under the supervision of a CF physiotherapist on the first evening post-operation. Before the operation, she had received continuous intravenous antibiotics for 41 days and these were continued for a further 6 days postoperatively until she was discharged. Histopathology of the resected segment of colon revealed a Stage I (T1N0) moderately differentiated adenocarcinoma with clear resection margins. Adjuvant therapy was not considered appropriate. She has remained very well over the 12 months since her return to Tasmania and a restaging CT scan of the abdomen and colonoscopy have shown no evidence of recurrence.

## Discussion

Our case illustrates a number of difficult management decisions. The patient had severe lung disease and was considered unlikely to survive an open laparotomy. The consensus opinion was that a laparoscopic approach would be associated with less pain and allow her to mobilise and perform airway clearance soon after extubation. There were still concerns that the high omental dissection required would cause discomfort, but this did not eventuate. The second and perhaps most important consideration was whether she would remain a potential lung transplant candidate even if she underwent a potentially curative procedure. An argument was made not to proceed with high-risk bowel surgery if the history of colonic malignancy precluded later transplantation. Immunosuppression post-lung transplant increases the risk of primary malignancy and the likelihood of recurrence of previous tumours. However, the risk of bowel cancer remains predominantly anecdotal. A large retrospective study of 3595 solid organ transplants performed over a 10-year period found an increased incidence of anal but not colorectal cancers compared to age-matched, non-transplanted patients [6]. A further study in 150 patients who developed colorectal cancer de novo following solid organ transplants found that these patients were younger and had a significantly poorer 5-year survival than non-transplanted patients with the same diagnosis [7]. This survival difference was most marked for Stage III and IV tumours, but was evident even for Stage I. However, the prognosis for our patient with a Stage I (T1) tumour should be excellent with the risk of recurrence over 5 years reported as being somewhere between 3% and 7% [8]. Although no guidelines exist with respect to lung transplantation in the setting of complete resection of a Stage I colonic malignancy, the current advice from the transplant unit is that the patient remains a potential candidate for lung transplantation, given her very early stage disease and apparent cure after 1 year's surveillance.

## Conclusion

A major determining factor in management decisions in this case was the patient herself. She was involved in all discussions and was fully aware of her operative risk and chose to undergo the laparoscopic procedure. She considered lung transplantation to be a separate issue. Interestingly, she was fully prepared to die from CF lung disease having faced this prospect her entire life, but did not want to die from a bowel obstruction or disseminated malignancy.

Our case illustrates the complexity of management decisions for patients with advanced CF lung disease and malignancy. Patients with severe CF lung disease may successfully undergo laparoscopic resection of bowel malignancies and as CF survival continues to improve, there may be an increasing demand for such procedures. The other major consideration to be addressed in transplant guidelines is the duration of surveillance needed before transplantation can be undertaken confidently following successful resection of a Stage I colon cancer.

## Consent

Written informed consent was obtained from the patient for publication of this case report. A copy of the written consent is available for review by the Editor-in-Chief of this journal.

## Competing interests

The authors declare that they have no competing interests.

## Authors' contributions

ANL was the Advanced Respiratory Trainee and DWR was the Cystic Fibrosis specialist, involved in this patient's care at the Royal Hobart Hospital,. ANL acquired this patient's perioperative records from the interstate hospital, and, having performed a literature review, drafted the manuscript. DWR revised the manuscript critically for important intellectual content. ANL replied to the Peer Reviewers' comments. Both authors read and approved the final manuscript.
